# Early Flowering and Maturity Promote the Successful Adaptation and High Yield of Quinoa (*Chenopodium quinoa* Willd.) in Temperate Regions

**DOI:** 10.3390/plants13202919

**Published:** 2024-10-18

**Authors:** Nazgol Emrani, Nathaly Maldonado-Taipe, Mario Hasler, Dilan S. R. Patiranage, Christian Jung

**Affiliations:** 1 Plant Breeding Institute, Christian-Albrechts-University of Kiel, Olshausenstr. 40, 24098 Kiel, Germany; nathaly.maldonado@ikiam.edu.ec (N.M.-T.); dilan.patiranage@kws.com (D.S.R.P.); c.jung@plantbreeding.uni-kiel.de (C.J.); 2 Applied Statistics, Christian-Albrechts-University of Kiel, Hermann-Rodewald-Straße 9, 24098 Kiel, Germany; hasler@email.uni-kiel.de

**Keywords:** yield, yield components, quality traits, adaptation, stability analysis, selection index, grand mean difference

## Abstract

Quinoa (*Chenopodium quinoa* Willd.) can offer an alternative for staple food considering its tolerance to abiotic stresses and high seed quality. However, its cultivation in temperate regions has not been successful due to its photoperiod sensitivity and low seed yield. This study investigated the agronomical performance and quality traits of 48 accessions for cultivation in northern Europe. We conducted two-year field trials and phenotyped traits related to phenological development, plant architecture, yield components, seed quality, and disease resistance. The major determinants of seed yield in this study were days to flowering, days to maturity, thousand-kernel weight, and panicle density, while downy mildew susceptibility and stem lodging showed a negative correlation with seed yield. We developed a selection index to enable simultaneous selection based on different important agronomical traits. We evaluated the stability of different accessions over the two years of the experiment. Finally, we provided a list of 10 selected accessions that can be directly integrated and serve as new crossing parents in quinoa breeding programs for temperate regions.

## 1. Introduction

Quinoa (*Chenopodium quinoa* Willd.) is a pseudocereal native to the Andean region of South America. It is a major source of staple food in the Andean region, where, among other uses, its seeds are used to make flour, soup, cereal, and alcohol [1]. Quinoa exhibits resistance to insects and diseases and tolerance to frost, drought, and salinity [2]. Moreover, quinoa seeds have a high protein content and balanced amounts of lysine and eight other essential amino acids, which makes them a suitable alternative for meat in vegetarian and vegan diets [3]. Considering the facts mentioned above, quinoa can provide a solution to meet the growing demand for high-quality food production, not only in marginal regions not suitable for the cultivation of major crops but also in temperate regions facing unpredictable weather conditions due to climate change. Quinoa production, trade, and consumption worldwide have grown rapidly because of high consumer demand for healthy, nutritious, and gluten-free food products. Interest in quinoa cultivation has also grown because of its remarkable adaptability to extreme climatic and soil conditions [4]. However, despite the promising aptitude of quinoa as a staple food, the production of quinoa is mainly limited to Peru, Bolivia, and Ecuador. Quinoa production worldwide was 147,038 tons in 2022, and Peru alone contributed to more than 75% (113,376 tons) of its global production (FAOSTAT, June 2024). Quinoa was first introduced to Europe in the 1970s [5]. However, commercial production started in 2016, and the crop yield was initially low [6]. Therefore, breeding attempts to increase yield potential have started in temperate regions.

Introducing new germplasm into different geographical regions requires adaptation to diverse environmental conditions, mainly regulated through photoperiod sensitivity and flowering time. Quinoa is generally known as a short-day species and, therefore, is sensitive to photoperiod in all its developmental stages, particularly during the seed-filling and maturity stages [7]. A day length of more than 12 h, which is the case during the cultivation season in Northern Europe, leads to continued vegetative growth and flowering and inhibits maturity [8]. High photoperiodic sensitivity and, as a consequence, low yield, are the major factors that limit quinoa cultivation in regions outside its center of origin [9].

Quinoa accessions are divided into two main groups (1) the coastal type from southwestern Chile and (2) the Andean highland type from southern Colombia to northwestern Argentina based on different independent studies using molecular markers [10,11,12]. A broad genetic variation was previously reported in both groups [13], although Patiranage et al. [14] reported a higher genetic diversity of the highland population based on the more comprehensive analysis of the sequencing data from a diversity panel of quinoa. Nevertheless, quinoa varieties with high seed yield and good quality for use in human food and industry developed by different breeding programs in temperate regions were driven from a small number of varieties bred by local breeders for higher yield and yield components mainly in the coastal regions of southern Chile, with similar environmental conditions like northern Europe [15] representing a very narrow genetic base for quinoa breeding programs [16]. Therefore, efforts should be concentrated on introducing new germplasm in the breeding programs through crosses to increase the genetic diversity of quinoa accessions [17].

A few studies already reported on the performance of quinoa accessions in temperate regions. One of these studies evaluated the agronomic performance of 13 quinoa varieties under north–west European field conditions during three growing seasons and reported a yield ranging from 0.47 to 3.42 t/ha, with late-maturing accessions showing the lowest seed yield [18]. Another study found low yield and protein content stability of four European quinoa cultivars in two consecutive growing periods in Southwestern Germany [19]. These studies found that the best yield- and quality traits were not combined in one cultivar, making breeding for adapted quinoa varieties crucial.

Despite the long history of this crop and worldwide interest in its cultivation, quinoa breeding is still in its infancy. The main breeding aim for quinoa in temperate regions is to develop high-yielding varieties with high protein and low saponin content [4,20]. Saponins are bitter-tasting triterpenoid glucosides found on the outer seed coat of quinoa seeds. Saponins reduce the digestibility and palatability of quinoa seeds and are, therefore, undesirable for human consumption and should be removed before consumption [21]. Moreover, the protein content and amino acid composition of the accessions considered for breeding should be improved, given the potential of quinoa as an alternative protein source in human nutrition. Furthermore, among essential amino acids, increasing leucine and lysine content should be specifically considered to meet the daily essential amino acid requirements in diets [22].

From an agronomic perspective, shorter non-branching plants with compact panicles and increased tolerance to abiotic and biotic stresses are desired. Another important breeding goal is to create photoperiod-insensitive varieties more suited to local conditions. Because quinoa is a short-day species, it must be adapted to long-day conditions when grown in temperate climates and high latitudes such as northern Germany [14]. An obstacle to growing quinoa in the humid environmental conditions of northern Europe is the downy mildew susceptibility. The oomycete downy mildew (*Peronospora variabilis*) is the most important pathogen of quinoa, causing severe yield losses of up to 99% [23].

Improvement in more than one trait at a time is required to develop a new variety. However, considering the epistatic interactions between loci regulating different traits, improving one trait might result in the deterioration of other related traits. Therefore, all agronomically important traits should simultaneously be considered in a breeding program. Selection indices provide a reliable strategy for the simultaneous selection of different traits. They provide a linear function of different traits weighted by an index coefficient [24]. Among other suggested coefficients, the heritability of the traits can be used for constructing a simple linear phenotypic selection index, where traits with higher heritability would have a more robust representation in the selection index and, therefore, are more extensively considered for selection [25]. The simultaneous selection of superior genotypes based on multiple agronomic traits using a selection index has been reported in several previous studies [26,27,28].

Quinoa can provide a solution to meet the growing demand for high-quality food due to its outstanding nutritional value and its strong tolerance against abiotic stress factors like drought and salinity. Quinoa cultivation in temperate regions can diversify the agricultural landscape and enhance agricultural sustainability. Moreover, considering the increasing demand for gluten-free and plant-based products in Europe, quinoa can offer an alternative as a plant-based source of protein and essential amino acids. Furthermore, the increasing temperatures and lower precipitation in spring observed in Europe over the last few years call for introducing new resilient crops into cropping systems. However, quinoa cultivation in temperate regions requires adaptation to shorter growing seasons, long days, and cooler temperatures. Therefore, it is important to investigate quinoa accessions in replicated experiments in these regions to identify the putative-adapted accessions that can thrive in this novel environment.

This study aimed to identify adapted quinoa accessions for cultivation in temperate regions by testing their performance under field conditions for two consecutive years in northern Germany. Using grand mean analysis and selection indices, we identified 10 accessions suitable for cultivating in temperate regions.

## 2. Materials and Methods

### 2.1. Plant Material, Experimental Design, and Phenotyping

For this experiment, 48 accessions were selected from previously established a core collection [14], where 310 quinoa accessions were investigated for agronomical traits in northern Germany over two years (Table 1). These accessions matured earlier than 160 days after sowing in northern Germany [14]. The accessions were grown from April to September in 2020 and 2021 near Traventhal, northern Germany (53°54′03.3″ N 10°19′47.8″ E), in 1.5 × 8.5 m^2^ plots in a complete randomized block design with column effects and three repetitions (Supplementary Figure S1). We used a sowing density of 80 seeds/m^2^ and a sowing depth of 1 cm. As fertilizer, 145.7 kg/ha nitrogen, 25.2 kg/ha P_2_O_5,_ 120 kg/ha K_2_O, and 33.2 kg/ha sulfur were applied before sowing. Accessions were sown in four columns within each block, and accessions expected to be ready to harvest simultaneously were clustered together in the same columns. The plots were mechanically harvested by a combine harvester 18 to 21 weeks after sowing. We applied mechanical harvest when the panicle turned brown, and the stem remained green with about 20% seed moisture.

To evaluate the performance of the 48 accessions in the field, we phenotyped days to flowering, field emergence, homogeneity, panicle length, plant height, panicle density, panicle shape, stem lodging, downy mildew susceptibility, saponin content, thousand kernel weight (TKW), and seed yield (t/ha) based on the published guidelines [29,30] (Supplementary Table S1).

### 2.2. Statistical Analysis

The statistical software R version 4.3.3. [31] was used for the statistical evaluation of the data. It consisted of the three following steps:

#### 2.2.1. Analysis for the Single Traits

For each trait, a separate appropriate mixed model [32] was defined, as follows:

(1) $$y_{ijk}=\mu +g_{i}+a_{j}+{\left(ga\right)}_{ij}+b_{k}{+e}_{ijk}$$

where, $y_{ijk}$ is trait observation; μ is the total mean of the population; $g_{i}$ is the fixed accession effect (*i* = 1, …, 48); $a_{j}$ is the fixed year effect (*j* = 1, 2); ${\left(ga\right)}_{ij}$ is the fixed interaction effect of accession and year; $b_{k}$ is the random block effect (*k* = 1, 2, 3); and $e_{ijk}$ refers to the residuals.

The residuals $e_{ijk}$ were assumed to be normally distributed, heteroscedastic (if necessary and possible), and correlated due to the years. These assumptions are based on a graphical residual analysis. Based on this model, a pseudo R^2^ was calculated [33], and an analysis of variances (ANOVA) was conducted. Afterward, multiple contrast tests [34,35] were used to compare each accession with the total average (grand mean) per year and the two years for each accession. Based on these tests, the corresponding grand mean differences were calculated for each accession in both years. The corresponding results allowed the identification of the accessions that performed significantly better than an “average accession”. Moreover, stable accessions throughout experiments in both years could be identified in this way. Additionally, a Pearson’s correlation analysis was performed for the traits, split for the two years.

#### 2.2.2. Heritability

For each trait, the heritability ($h^{2}$) was calculated based on the following random effects model:

(2) $$y_{ijkl}=\mu +g_{i}+a_{j}+{\left(ga\right)}_{ij}+b_{k}+{\left(ba\right)}_{kj}+{c_{l}+e}_{ijkl}$$

where $y_{ijkl}$ is the trait observation; μ is the total mean; $g_{i}$ is the random accession effect (*i* = 1, …, 48); $a_{j}$ is the random year effect (*j* = 1, 2); ${\left(ga\right)}_{ij}$ is the random interaction effect of accession and year; $b_{k}$ is the random block effect (*k* = 1, 2, 3); ${\left(ba\right)}_{kj}$ is the random interaction effect of block and year; $c_{l}$ is the random column effect (*l* = 1, …, 48); and $e_{ijkl}$ refers to the residuals.

In contrast to the model (1), the correlations between the data produced in each year were not modeled directly, but some additional random effects were considered in this model. Modeling the residuals’ correlations and appropriate random effects can be used here, but random factors must be considered when calculating heritability. The following heritability formula was used:

(3) $$h^{2}=s_{g}^{2}/(s_{g}^{2}+s_{ga}^{2}/2+s_{r}^{2}/6)$$

where $s_{g}^{2}$ is the accession variance-, $s_{ga}^{2}$ is the variance of accession × year, and $s_{r}^{2}$ is the residual variance.

#### 2.2.3. Selection Index

For each accession, the selection index (I) was calculated based on the most important agronomical traits for the selection of adapted lines; thousand kernel weight, seed yield, days to flowering, plant height, downy mildew susceptibility, and saponin content using the following equation [21]:

(4) $$I=\sum_{m}h_{m}^{2}\tilde{y_{m}}$$

where the heritability is $h_{m}^{2}$, and the standardized trait values $\tilde{y_{m}}$ were used for *m* = thousand kernel weight, seed yield, days to flowering, plant height, downy mildew susceptibility, and saponin content. The $\tilde{y_{m}}$ values had been multiplied by minus one, if necessary, so that higher index values always represent a desired effect for each trait. The selection index was then considered an additional trait, for which the same statistical evaluation as described under Section 2.2.1 was conducted.

## 3. Results

### 3.1. Phenotypic Analysis of Quinoa Accessions under Field Conditions

We observed a substantial variation in all the investigated traits. The earliest flowering accession flowered 16 days earlier than the latest one (Table 2). Two of the most variable traits recorded in this study were panicle length and plant height, where a difference of 52.5 cm and 105 cm was observed between the shortest and tallest accessions, respectively. Moreover, we observed a huge variation in seed yield and TKW (Table 2).

The analysis of variance (ANOVA) revealed statistically significant differences between the accessions for all the investigated traits (*p* < 0.001). Moreover, we observed a significant difference between the years for emergence, plant height, panicle length and density, stem lodging, saponin content, and seed yield, whereas DTF, homogeneity, panicle shape, downy mildew susceptibility, and TKW were not different between years. However, all traits were significantly affected by accession × environment interactions (Table 3). We estimated a moderate to high heritability for all traits, except for panicle length (h^2^ = 0.2) and panicle shape (h^2^ = 0.29).

**Figure 1:**
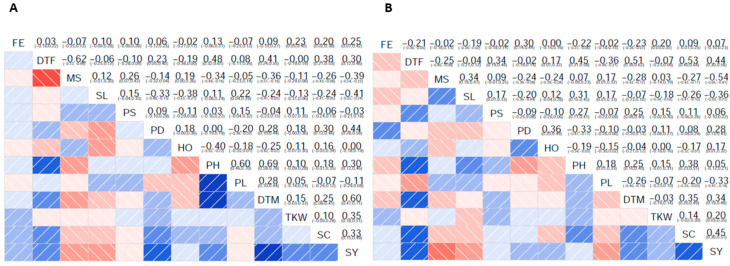
Pearson’s correlation coefficients (and corresponding 95% confidence intervals) between the traits in 2020 (**A**) and 2021 (**B**). FE: field emergence, DTF: days to flowering, MS: downy mildew susceptibility, SL: stem lodging, PS: panicle shape, PD: panicle density, HO: Homogeneity, PH: plant height, PL: panicle length, DTM: days to maturity, TKW: thousand kernel weight, SC: saponin content, SY: seed yield.

Generally, Pearson’s correlation coefficient between the traits was comparable in both years. As expected, DTF and DTM showed significant positive correlations with each other and PH in both years. Interestingly, we observed a significant negative correlation between DTF and DTM with downy mildew susceptibility in both years. Moreover, later flowering and maturing accessions tended to have a higher saponin content. In both years, seed yield was positively correlated with TKW, DTM, and DTF, while downy mildew susceptibility and stem lodging significantly negatively affected seed yield. Furthermore, high-yielding accessions tended to have more saponin in their seeds in both years (Figure 1).

### 3.2. Calculation of a Selection Index Based on Important Agronomical Traits

We calculated the selection index for simultaneous selection based on TKW, seed yield, days to flowering, plant height, downy mildew susceptibility, and saponin content for each year. These traits were considered for calculating the selection index since they are the most important agronomical traits to be considered for the successful cultivation of quinoa in northern Europe. Moreover, these traits show a moderate to high heritability, which makes them suitable for selection under different environmental conditions/years. The Chilean accessions NL-6 and BO-32 showed the highest and the lowest selection index in both years, respectively (Table 4). Moreover, seven accessions were found among the 10 best accessions ranked based on the selection index in both years of the experiment. The selection index was mainly comparable between the years for each accession. However, we observed a contrasting ranking for a few accessions in 2020 compared to 2021. Accessions Bouchane-3 and Ames-13721 had a considerably higher selection index in 2020 compared to 2021, while PI-614927 had a noticeably better ranking based on the selection index in 2021 compared to 2020.

To select the most stable accessions among the years, we compared the selection index between the years for every accession. Our result showed that for 25 accessions, there was no significant difference between the selection indexes in 2020 compared to 2021 (Table 5). Therefore, these accessions could be considered stable accessions.

### 3.3. Selection of Adapted Quinoa Accessions for Cultivation in Temperate Regions

In the last step, we calculated the grand mean differences per year for days to flowering, downy mildew susceptibility, plant height, TKW, saponin content, seed yield, and selection index. Suitable accessions for northern Europe should be short and flower early, display lower downy mildew susceptibility, and have a low saponin content. Moreover, their TKW and seed yield should be high in combination with a high selection index. Most of the earliest flowering accessions showed significantly shorter days to flowering compared to the mean in both years based on the grand mean differences analysis (Supplementary Table S2A). However, Titicaca (QP-346) flowered later compared to the mean in the first year but earlier in the second year, possibly due to higher precipitation in 2021 compared to 2020. For plant height, we observed the same contrasting results for accession Moroccan Yellow (QP-002) (Supplementary Table S2B). Furthermore, accession RU-5 (QP-035) showed higher seed saponin content compared to the mean in 2020, but lower levels in 2021 (Supplementary Table S2C). Accession PI-614889 (QP-004) showed significantly higher TKW in 2020 but lower levels compared to the mean in 2021 (Supplementary Table S2E). Consistent results across both years were obtained for downy mildew susceptibility, seed yield, and selection index (Supplementary Table S2).

In both years, seven accessions flowered earlier compared to the population mean (Supplementary Table S2A). Likewise, five accessions were significantly shorter in both years (Supplementary Table S2B). The Chilean accession Nde-09 (QP-086) depicted a significantly higher TKW and seed yield, which was also confirmed in the second year of the experiment (Supplementary Table S2E,F). This accession also showed a significant improvement in the selection index compared to the population mean in both years (Supplementary Table S2G).

Finally, based on the grand mean difference analysis, we identified 10 stable early-flowering accessions with higher seed yield, lower downy mildew susceptibility, and saponin content better adapted to northern Germany’s temperate regions (Table 6 and Supplementary Figure S2). Four accessions originated from the lowlands of Chile and two resulted from a Danish breeding program.

## 4. Discussion

Our study aimed to assess the adaptation of quinoa accessions to temperate regions. We investigated these accessions’ performance in two years and identified 10 accessions that can be considered for cultivation in temperate regions. These accessions not only produce a comparatively high yield in northern Europe but also have a higher downy mildew susceptibility which is one of the most important factors for the successful cultivation of quinoa in humid regions of northern Europe. Moreover, these accessions have lower seed saponin content, increasing quinoa’s palatability for human consumption.

We selected 48 accessions based on a previous study [14], where days to maturity were the main determining factor for the selection of accessions for cultivation in northern Europe. Therefore, based on the results of this study, we selected early maturing accessions (DTM ≤ 160) with a potential for cultivation in northern Europe. There was a significant accession × environment interaction for all the investigated traits, probably due to differences in weather conditions between the years. While minimum and maximum temperatures were comparable between both years, there was much higher precipitation in 2021 (386.4 mm) compared to 2020 (233.1 mm), specifically during the seed-filling stage between the end of June and the beginning of August (130.5 mm in 2021 compared to 92.6 mm in 2020) (Supplementary Figure S3, Supplementary Table S5). This possibly had a positive effect on seed yield, as the mean seed yield over all accessions was higher in 2021 (3.08 t/ha) compared to 2020 (2.13 t/ha), although we did not observe any significant difference between the means of other yield-related traits in 2021 compared to 2020. We decided to analyze the data for each year separately to better estimate the effects of the environments on all phenotypes. Apart from days to flowering, all the other investigated traits showed considerable variation, which can be exploited in breeding programs. The narrow genetic variation for days to flowering was expected, as we considered only early flowering and maturity accessions for this study. Furthermore, we recorded relatively high TKW and seed yield for some of the accessions, which shows the potential of the selected accessions for cultivation in northern Europe. Moreover, we observed medium to high heritability for all the investigated traits apart from panicle length and shape. Previous studies also reported low to moderate heritability for panicle length [36,37]. This indicated that most of the phenotypic variation observed in the investigated traits in this study is caused by the genotype and therefore, can be improved by breeding.

We observed that later flowering accessions were, on average, taller and reached maturity later than early ones. This observation is in line with previous studies [14,36,38]. Therefore, the selection of early accessions would facilitate the development of a quinoa ideotype for temperate regions. Additionally, we observed a significant positive correlation coefficient between days of flowering and saponin content in both years (Figure 1). Oustani et al. [38] also reported similar results, unlike other reports, which found no significant correlations between saponin content and morphological traits [14,36,39]. Since reducing saponin content is an important breeding objective, selecting early flowering accessions could potentially lead to lower seed saponin content accessions.

Interestingly, DTF and DTM showed a significant positive correlation with seed yield in both years of the experiment. This relationship was also reported in a previous study [40]. However, the opposite is usually reported for quinoa [14,36]. It is important to note that all the accessions investigated in this study have early to moderate days to flowering. A very early flowering phenotype may have a negative penalty on seed yield, as early flowering reduces the time available for sufficient carbon assimilation and causes insufficient growth of photosynthetic organs during vegetative development to guarantee high seed yield [41,42]. Remarkably, the considerable variation for TKW and seed yield reported in this study suggests that early flowering alone will not guarantee a higher yield of accessions in temperate regions. Moreover, very early flowering and maturing-accessions were more susceptible to downy mildew in both years (Figure 1). This indicates that relatively late flowering accessions are more resistant, putatively due to an “escape” mechanism due to their slower phenological development. However, since we pre-selected the plant material in this study for earliness, we recommend investigating the relationship between these traits in further experiments with a more diverse panel of quinoa accessions under different environmental conditions for confirmation.

The major determinants of seed yield with the highest positive correlation coefficient, apart from DTF and DTM, were TKW and panicle density, while downy mildew susceptibility and stem lodging, as expected, affected seed yield negatively (Figure 1). TKW is a major determinant of quinoa seed yield, and a positive correlation between this trait and seed yield was expected. Moreover, increased panicle density would lead to more seeds, potentially increasing seed yield. This was also in line with previous reports in quinoa [14].

We used grand mean difference analysis to find superior accessions in this study. Our results showed that accession EMBRAPA-Brazil was, on average, significantly shorter and had a higher TKW than the population mean in both years, leading to a significantly higher selection index than the population mean for this accession (Table 6). We observed the same trend for accession Nde-09, which, together with accessions NL-6 and BO-63, also depicted a significant grand mean difference for seed yield throughout the experiment. BO-63 was also significantly less susceptible to downy mildew than the population mean, showing its great potential for cultivation in northern Germany.

Based on our previous study [14], only ICBA-Q3 belongs to the highland population. It has already been shown that most of the high-yielding quinoa accessions bred for temperate European regions are derived from the coastal (lowland) population, mostly from Chile [43]. Therefore, integrating the highland accession identified in this study as a crossing parent into breeding programs can diversify quinoa germplasm in temperate regions.

A previous study in our group identified maker-trait associations for agronomical traits in a diversity panel of quinoa using a genome-wide association study [14]. As a result of this study, three genes were found in the vicinity of associated SNPs for seed weight, plant height, and flowering time, and their haplotype variation was studied. The most significant SNP associated with DTF, DTM, PH, and PL was located within the *CqGLX2-2* gene, which encodes an enzyme from the glyoxalase family. It was shown that cytosine at this SNP position is associated with early flowering, early maturity, short panicles, and short plant height, which are desirable traits for the cultivation of quinoa in temperate regions. Based on the published sequencing data [14], we identified the haplotypes of the 10 best accessions at this locus (Supplementary Table S3). Seven of nine accessions for which high-quality sequencing data were available for this locus showed a cytosine at this SNP position. Moreover, Patiranage et al. [14] found that accessions carrying the *PP2C* haplotype 3 and *RING* haplotype 7 produced larger seeds than accessions carrying other haplotypes. We observed that most of the selected accessions in this study carried the beneficial haplotypes of these two genes (Supplementary Table S3). These results confirm the results of the previous study that the loci mentioned above should be selected in quinoa breeding programs in temperate regions. Apart from this study, two other studies in our group identified QTL for agronomically important traits [36] and several differentially expressed genes in response to photoperiod [44]. We recommend investigating the genotypes of the selected accessions for the candidate loci identified in those studies and developing molecular markers for these loci for marker-assisted selection in quinoa.

Given the potential of quinoa as a promising source of protein in human nutrition in the future, it is also important to investigate and improve the protein content and amino acid composition of the accessions considered for breeding. A recent study reported the variation in different seed quality traits in 360 different accessions in quinoa [45]. Apart from one accession, all the other accessions investigated in our study were also present in this study. Therefore, we compared the quality traits of our accessions based on this study (Supplementary Table S4). We observed a wide range of the investigated traits in our panel. Moreover, the 10 selected accessions in this study showed a medium to high protein content compared to the other investigated accessions. This offers a promising perspective when considering quinoa as an alternative protein source for vegetarian and vegan diets. Considering the essential amino acids leucine and lysine, among the accessions investigated in this study, Brightest-Brilliant-Rainbow, Ames-13745, Redhead and Cherry-Vanilla with more than 60 mg/g protein lysine, and Bo-51 and Moroccan Yellow with 62.5 mg/g protein) leucine offer a promising potential for improving protein quality in the human diet. Considering the selection criteria in the current study, these accessions were not among the 10 selected accessions. However, they can be considered for crosses with the selected accessions to provide new genetic variation by combining improved agronomical and quality traits.

We suggest testing the agronomical performance and quality traits of the selected accessions in this study in diverse environmental conditions in temperate regions of Europe to confirm their suitability for integration into breeding programs in these regions. Furthermore, earlier sowing dates (in March) and increased sowing density should be tested in further trials, as the fast-early establishment would help quinoa to compete with weeds and offer a solution for one of the main practical obstacles of quinoa cultivation. In our study, mechanical sowing and harvest were perfectly possible with minor adjustments of sowing machines and combines typically used for cereals. Moreover, we observed that mechanical harvest is possible even for quinoa with a seed moisture of around 20%, much above the recommended seed humidity of 12% at harvest for cereals. However, further assessment of the energy consumption and costs of drying seeds after harvest should be considered to ensure the sustainable production of quinoa in temperate regions. Furthermore, considering the effects of climate change, further traits like drought and heat tolerance and early establishment should be considered as breeding objectives in quinoa breeding programs.

This study investigated the agronomical and quality performance of 48 quinoa accessions for cultivation in temperate regions and identified 10 promising accessions for cultivation in northern Europe. The accessions identified in this study will lay the foundation for quinoa breeding programs in temperate regions and they have already been used as crossing parents in our breeding program, from which advanced F_5_ lines are available. The selected lines considering important agronomical and quality traits can be introduced as new cultivars to the market and integrated into the crop rotation in temperate regions to diversify the cropping systems in these regions.

## Figures and Tables

**Table 1:** Accessions used in this study and their origins.

Seed Code	Accession Code	Accession Name	Origin
195120	QP-002	Moroccan Yellow	NA
195121	QP-003	Bouchane-3	NA
195122	QP-004	PI-614889	Chile
195123	QP-005	ICBA-Q5	NA
195124	QP-006	PI-614927	Bolivia
195125	QP-019	E-DK-4	NA
195126	QP-026	Indian Quinoa	NA
195127	QP-030	PUC-mix-red	Chile
195128	QP-032	Brightest-Brilliant-Rainbow (BBR)	NA
195129	QP-035	RU-5	United Kingdom
195130	QP-036	Regalona	Chile
195131	QP-041	Ames-13721	United States
195132	QP-042	Ames-13745	United States
195133	QP-043	Oro-de-Valle	NA
195134	QP-046	Ames-13744	United States
195135	QP-055	PUC-mix-green	Chile
195136	QP-060	Ames-13743	Chile
195137	QP-061	BO-58	Chile
195138	QP-065	Vikinga	NA
195139	QP-084	EMBRAPA-Brazil	NA
195140	QP-086	Nde-09	Chile
195141	QP-089	RU-2	United Kingdom
195142	QP-096	PI-634923	Chile
195143	QP-097	NSL-86649	NA
195144	QP-099	BO-29	Chile
195145	QP-103	BO-03	Chile
195146	QP-105	NL-6	Chile
195147	QP-107	BO-32	Chile
195148	QP-108	BO-31	Chile
195149	QP-113	Redhead	NA
195150	QP-126	BO-30	Chile
195151	QP-127	Bouchane-4	NA
195152	QP-128	PI-614883	Argentina
195153	QP-139	NSL-91567	NA
195154	QP-141	PI-634921	NA
195155	QP-165	BO-51	Chile
195156	QP-169	D-11889	Argentina
195157	QP-172	PI-634919	Chile
195158	QP-175	BO-63	Chile
195159	QP-176	BO-42	Chile
195160	QP-181	BO-11	Chile
195161	QP-220	PI-634918	Chile
195162	QP-225	Cherry-Vanilla	United States
195163	QP-231	Bouchane-2	NA
195164	QP-232	Bouchane-1	NA
195165	QP-233	ICBA-Q3	NA
195166	QP-343	PI-614886	Chile
195167	QP-346	Titicaca	Denmark

NA: not available.

**Table 2:** Variation in the traits measured over a two-year field cultivation in 48 quinoa accessions. DTF: days to flowering, TKW: thousand kernel weight, SD: standard deviation, CV: coefficient of variation.

Trait	Minimum	Maximum	Mean	SD	CV%
DTF	63	79	69.77	4.2	4.77
Emergence (%)	19.25	100	48.45	15.54	12.4
Homogeneity (%)	0	100	74.83	28.52	24.2
Panicle length (cm)	10	62.5	33.37	7.9	5.02
Plant height (cm)	112.5	217.5	160	20.56	8.44
Panicle density	1	7	4.71	1.23	17.05
Panicle shape	1	5	2.79	0.62	7.42
Stem lodging (%)	0	100	24.29	26.7	54.9
Mildew susceptibility	1	5	3.26	1.16	17.84
Saponin content (mm)	0	21	10.7	5.82	19.81
TKW (g)	1.48	4.9	2.42	0.35	10.86
Seed yield (t/ha)	0.18	5.8	2.61	1.03	27.27

**Table 3:** Analysis of variance and heritability of the traits phenotyped over two years in 48 quinoa accessions. Variance components were calculated using a random effect model, while analysis of variance (ANOVA) was performed using a mixed-effects model. DTF: days to flowering, TKW: thousand kernel weight, R^2^: coefficient of determination, h^2^: heritability, *** *p* < 0.001.

Trait	Variance Components				R^2^	h^2^
	Genotype	Year	Genotype × Year	Residual		
DTF	11.08 ***	0	5.94 ***	0.95	0.95	0.78
Emergence (%)	36.19 ***	51.95 ***	55.58 ***	119.16	0.62	0.43
Homogeneity (%)	327.45 ***	0	318.10 ***	155.44	0.8	0.64
Panicle length (cm)	2.80 ***	33.45 ***	11.85 ***	29.63	0.31	0.2
Plant height (cm)	181.49 ***	69.82 ***	135.87 ***	56.58	0.9	0.7
Panicle density	0.64 ***	0.41 ***	0.27 ***	0.34	0.77	0.77
Panicle shape	0.04 ***	0	0.14 ***	0.21	0.54	0.29
Stem lodging (%)	177.18 ***	56.49 ***	319.73 ***	171.27	0.77	0.48
Mildew susceptibility	0.34 ***	0	0.69 ***	0.31	0.78	0.46
Saponin content (mm)	4.43 ***	29.17 ***	5.48 ***	9.39	0.86	0.51
TKW (g)	0.06 ***	0	0.01 ***	0.02	0.99	0.86
Seed yield (t/ha)	0.51 ***	0.45 ***	0.11 ***	0.2	0.97	0.85

**Table 4:** Selection index and ranking of the accessions in 2020 and 2021.

Accession	Accession Name	I-2020	Ranking 2020	I-2021	Ranking 2021
QP-002	Moroccan Yellow	−1.31	29	−0.53	41
QP-003	Bouchane-3	2.38	3	0.71	28
QP-004	PI-614889	−0.66	18	−0.28	37
QP-005	ICBA-Q5	−1.2	24	−0.37	39
QP-006	PI-614927	−1.76	34	2.93	6
QP-019	E-DK-4	−2.07	40	−1.8	47
QP-026	Indian Quinoa	−1.85	37	1.8	13
QP-030	PUC-mix-red	−3.15	45	−0.3	38
QP-032	BBR	−1.8	36	−0.64	42
QP-035	RU-5	−1.03	22	0.77	26
QP-036	Regalona	−1.12	23	−0.42	40
QP-041	Ames-13721	1.06	7	−0.25	36
QP-042	Ames-13745	−1.71	33	−0.81	44
QP-043	Oro-de-Valle	−2.06	39	1.25	19
QP-046	Ames-13744	−1.77	35	1.05	21
QP-055	PUC-mix-green	−0.66	17	0.92	23
QP-060	Ames-13743	−0.03	13	2.07	11
QP-061	BO-58	−1.21	27	0.25	33
QP-065	Vikinga	1.72	4	2.28	9
QP-084	EMBRAPA-Brazil	0.88	9	3.48	3
QP-086	Nde-09	1	8	3.02	5
QP-089	RU-2	−1.48	30	1.97	12
QP-096	PI-634923	−1.53	31	0.77	25
QP-097	NSL-86649	−2.52	43	0.07	35
QP-099	BO-29	−0.92	19	2.29	8
QP-103	BO-03	1.35	6	2.53	7
QP-105	NL-6	2.49	1	4.31	1
QP-107	BO-32	−5.03	48	−2.81	48
QP-108	BO-31	−2.38	42	1.65	15
QP-113	Redhead	−1.59	32	0.73	27
QP-126	BO-30	−1.21	26	1.25	18
QP-127	Bouchane-4	0.66	10	1.52	16
QP-128	PI-614883	−2.64	44	0.69	30
QP-139	NSL-91567	−1.29	28	0.26	32
QP-141	PI-634921	0.34	11	1.76	14
QP-165	BO-51	−3.24	46	0.83	24
QP-169	D-11889	−1.2	25	1.01	22
QP-172	PI-634919	−4.04	47	−1.18	45
QP-175	BO-63	2.43	2	3.05	4
QP-176	BO-42	−2.22	41	0.62	31
QP-181	BO-11	−0.96	20	0.69	29
QP-220	PI-634918	−0.58	15	0.2	34
QP-225	Cherry-Vanilla	−0.99	21	−1.36	46
QP-231	Bouchane-2	−0.03	14	1.26	17
QP-232	Bouchane-1	0.25	12	1.11	20
QP-233	ICBA-Q3	−0.62	16	2.1	10
QP-343	PI-614886	−2.01	38	−0.78	43
QP-346	Titicaca	1.59	5	3.54	2

I: selection index.

**Table 5:** Absolute difference between the selection index values for each accession in 2020 and 2021, and the associated *p*-value. Multiple contrast tests were used to compare the selection index of each accession between the two years.

Accession	Difference	*p*-Value
QP-002	0.78	1.00
QP-003	1.67	0.99
QP-004	0.38	1.00
QP-005	0.82	0.39
QP-019	0.26	1.00
QP-032	1.16	0.49
QP-036	0.70	1.00
QP-041	1.30	1.00
QP-042	0.90	1.00
QP-055	1.58	0.45
QP-060	2.10	0.98
QP-061	1.46	0.15
QP-065	0.56	1.00
QP-103	1.19	1.00
QP-105	1.82	0.43
QP-127	0.85	1.00
QP-139	1.55	0.82
QP-141	1.42	0.76
QP-175	0.62	1.00
QP-181	1.65	0.87
QP-220	0.78	0.82
QP-225	0.37	1.00
QP-231	1.29	0.92
QP-232	0.86	0.69
QP-343	1.23	0.14

**Table 6:** List of the quinoa accessions suitable for cultivation in temperate regions based on the grand mean difference analysis.

Accession Code	Accessions Name	Origin	Days to Flowering		Downy Mildew Susceptibility		Height (cm)		TKW (g)		Saponin Content (mm)		Seed Yield (t/ha)		I	
			GMD-2020	GMD-2021	GMD-2020	GMD-2021	GMD-2020	GMD-2021	GMD-2020	GMD-2021	GMD-2020	GMD-2021	GMD-2020	GMD-2021	GMD-2020	GMD-2021
QP-003	Bouchane-3	NA	−6.98 ***	1.34	−0.28	0.73	−21.36 ***	15.65	0.02	0.34 ***	−9.16 *	3.84	0.26	0.29	3.29 **	−0.19
QP-065	Vikinga	Denmark	−4.96 ***	−5.63 ***	−0.08	0.39	−40.07 ***	−21.83 ***	−0.06	0.08	−11.19 **	−5.47	−0.48	−1.35	2.63 ***	1.37
QP-084	EMBRAPA-Brazil	NA	−0.96	−1.64	0.58	0.40	−37.36 ***	−24.43 ***	0.16 **	0.12 ***	5.51 ***	1.85	0.71	1.73 ***	1.79 ***	2.58 ***
QP-086	Nde-09	Chile	2.04 *	1.38 *	−0.76	−0.60	3.34	2.86	0.23 *	0.26 ***	0.52	0.50	1.75***	1.95 **	1.91 ***	2.12 **
QP-103	BO-03	Chile	−6.96 ***	−5.64 ***	2.08 ***	−0.94	−29.86 ***	−13.60	0.39 **	0.12 ***	−1.49	−3.82	−0.55 *	−1.11	2.26 ***	1.63
QP-105	NL-6	Chile	−6.96 ***	−5.65 ***	0.72	−1.26 *	−25.36	−24.30 ***	0.33 ***	0.13	2.18	−1.16	1.10 ***	0.73 *	3.40 ***	3.41 ***
QP-127	Bouchane-4	NA	−6.96 ***	−5.64 ***	0.91	1.06	−49.03 ***	−16.93	−0.08	−0.15 **	−3.49	−4.15 **	−1.32 ***	−0.64	1.57 ***	0.62
QP-175	BO-63	Chile	2.37 **	1.36	−1.92 ***	−2.27 ***	2.64	2.23	0.86 ***	0.48 ***	4.84	3.85	1.36 ***	0.70	3.34 ***	2.15 ***
QP-233	ICBA-Q3	NA	−4.94 ***	−1.64	1.78 ***	−0.96	20.74 ***	6.68	0.26	0.18 ***	0.85	−1.14	0.16 ***	0.01	0.30	1.20 *
QP-346	Titicaca	Denmark	2.05 *	−5.64 ***	−0.30	0.40	−30.49 ***	−28.19 ***	0.60 **	0.26 ***	−3.15	−5.81 ***	−0.28	−0.56	2.50 ***	2.64 ***

TKW: thousand kernel weight, I: selection index, GMD: grand mean difference, NA: not available, *: *p* < 0.05, **: *p* < 0.01, *** *p* < 0.001.

## Data Availability

Data is contained within the article or Supplementary Material.

## Supplementary Materials

The following supporting information can be downloaded at https://www.mdpi.com/article/10.3390/plants13202919/s1: Supplementary Table S1: Description of the methods used for phenotyping different traits in this study. Supplementary Table S2: Best performing accessions in each year based on the grand mean difference analysis for days to flowering (A), plant height (B), saponin content (C), downy mildew susceptibility (D), thousand kernel weight (E), seed yield (F), and selection index (G). Supplementary Table S3: Accession of the accessions for SNPs associated with seed weight, plant height, and flowering time. Haplotypes for the candidate genes are derived from a previous GWAS study [14]. Supplementary Table S4: Best linear unbiased estimates (BLUEs) for total protein and amino acid content of quinoa accessions investigated in this study based on [45]. Supplementary Table S5: The daily average temperature and precipitation for the experimental period in years 2020 and 2021 in Traventhal. Supplementary Figure S1: an aerial view from the field experiment taken in 2020 and the experimental design for the field experiments in 2020 and 2021. Supplementary Figure S2: Selected accessions for cultivation in northern Germany. Supplementary Figure S3: Minimum and maximum daily temperature (°C) (A) and cumulative precipitation (mm) (B) for Traventhal during the cultivation season in 2020 and 2021. Source: https://meteostat.net (accessed on 1 December 2021), Station: Wittenborn.
