# Supplementary material for: Early Flowering and Maturity Promote the Successful Adaptation and High Yield of Quinoa (Chenopodium quinoa Willd.) in Temperate Regions
Source: Plants (Basel). 2024 Oct 18;13(20):2919. doi: 10.3390/plants13202919 (PMC11511510; doi:10.3390/plants13202919)
Supplement: Supplementary file 1 [file plants-13-02919-s001.zip › plants-3227433-supplementary.pdf]

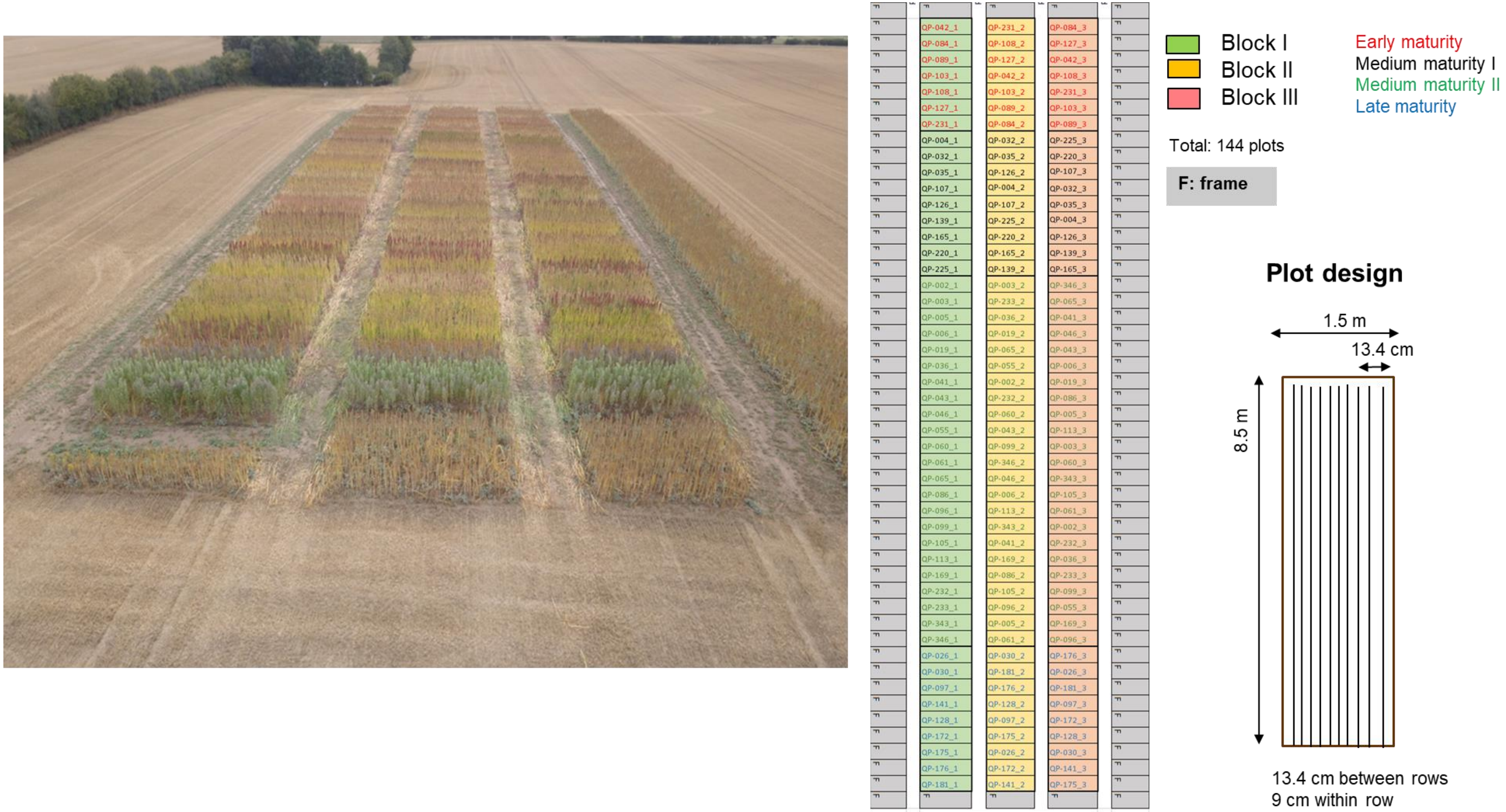

Supplementary Figure S1. An aerial view from the field experiment taken in 2020 and the experimental design for the field experiments in 2020 and 2021.

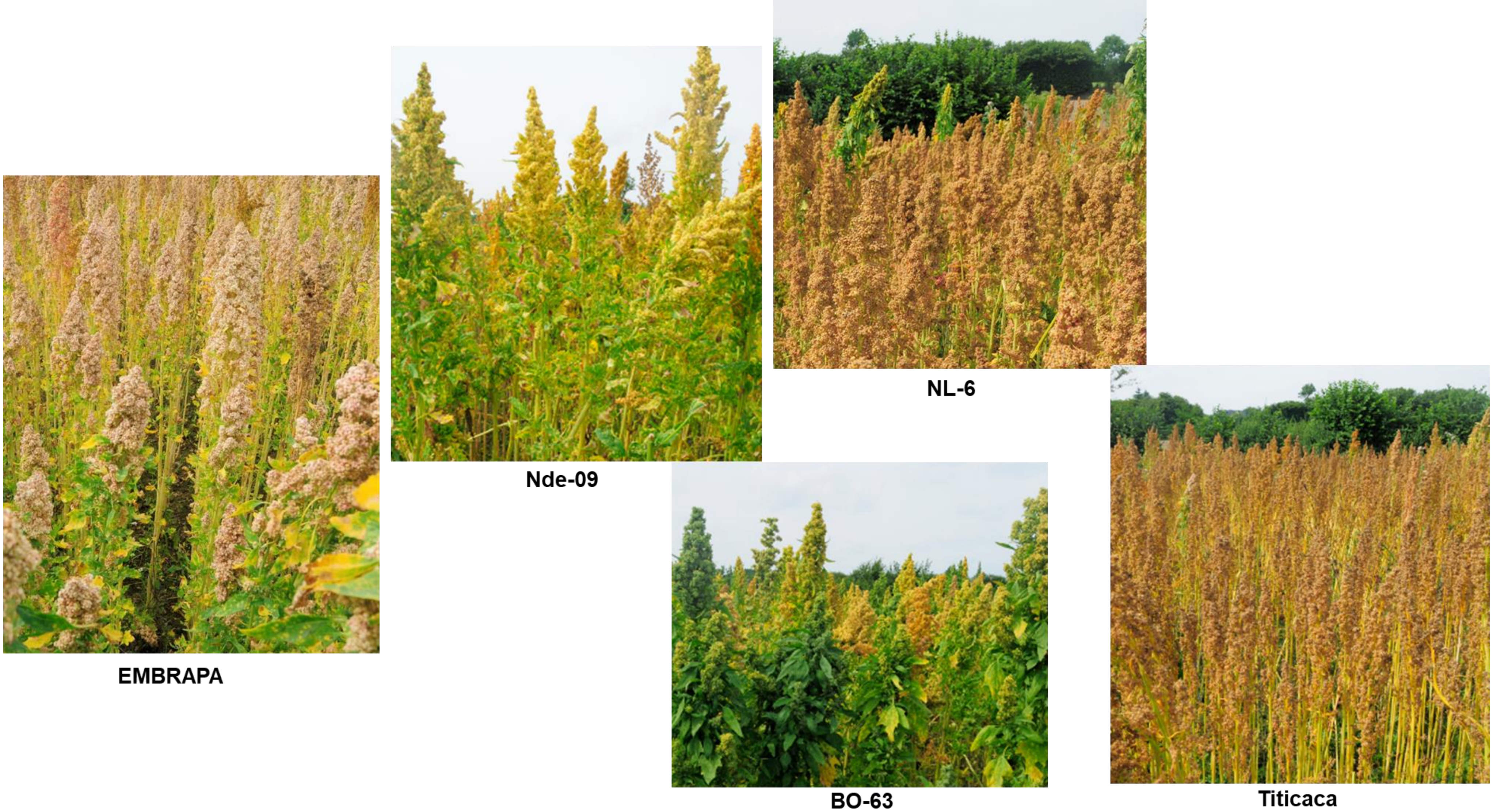

Supplementary Figure S2. Selected accessions for cultivation in northern Germany

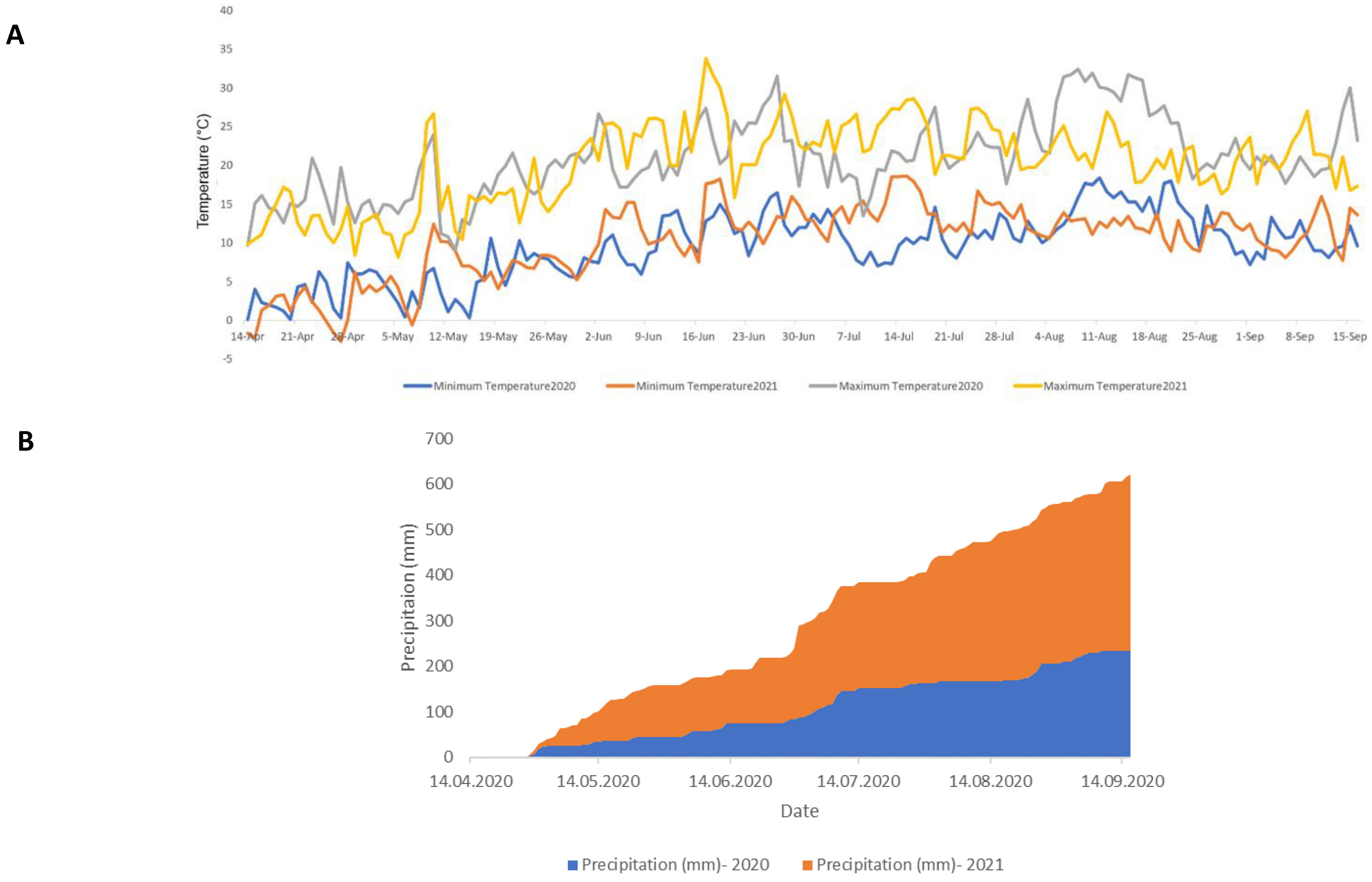

Supplementary Figure S3. Minimum and maximum daily temperature (°C) (A) and precipitation (mm) (B) for Traventhal during the cultivation season in 2020 and 2021. Source: https://meteostat.net, Station: Wittenborn

Supplementary Tables

Supplementary Table S1- Description of the methods used for phenotyping different traits in this study.

Phenotype	Abbreviation	Description
Field emergence (%)	FE	Number of plants in one lineal meter at the 2nd and 10th rows of each plot at BBCH 14
Days to flowering	DTF	Time of the first flower opening for 50% of the plants in each plot
Plant height*(cm)	PH	As the average height observed at two locations in each plot at mid seed filling stage BBCH 70
Panicle length* (cm)	PL	As the average length of panicles observed at two locations in each plot at mid seed filling stage BBCH 70
Homogeneity*(%)	HO	As approximate percentage of homogeneous plants in each plot. Homogeneity was defined by height, maturity stage, inflorescence color and panicle shape/density.
Stem lodging*(%)	SL	As approximate percentage of stem lodged plants in a plot at mid seed filling stage BBCH 70
Panicle density *	PD	Evaluated as scoring classes from 1 to 7: 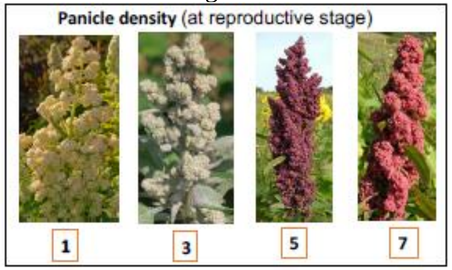
Panicle shape*	PS	Evaluated in a scale from 1 to 3: 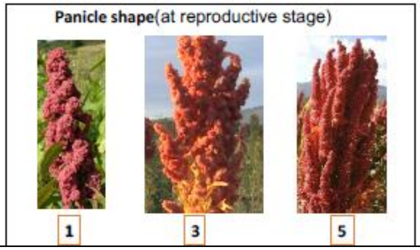
Days to maturity	DTM	90% of the panicles in each plot are completely brown (BBCH99)
Seed yield (t/ha)	SY	Seed harvest per plot at 12% humidity

Thousand kernel weight (g)	TKW	Weight of thousand seeds after overnight drying at 30 °C
Mildew susceptibility	MS	Scale from 1 to 5 (at flowering and at BBCH 70): 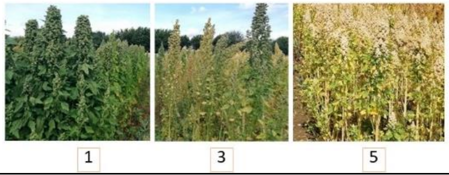
Saponin content (mm)	SC	As the height of foam formed by adding double distilled water to 5 seeds (foam method) (Koziol, 1991)

References:

Koziol, M.J. Afrosimetric estimation of threshold saponin concentration for bitterness in quinoa (*Chenopodium quinoa* Willd). Journal of the Science of Food and Agriculture 1991, 54, 211-219.

Supplementary Table S2- Best performing accessions in each year based on the grand mean difference analysis for days to flowering (A), plant height (B), saponin content (C), mildew susceptibility (D), thousand kernel weight (E), seed yield (F) and selection index (G).

A

**Accession**	**GMD-2020**	**GMD-2021**
QP-002	-5.6***	-0.6
QP-003	-7.0***	1.3
QP-041	-6.3***	1.4
QP-060	-7.0***	-5.6***
QP-065	-5.0***	-5.6***
QP-089	-2.3**	-5.6***
QP-103	-7.0***	-5.6***
QP-105	-7.0***	-5.6***
QP-127	-7.0***	-5.6***
QP-181	-7.0***	0.4
QP-225	-7.0***	1.4
QP-232	--7.0***	-5.6***
QP-233	-5.0***	-1.6
QP-346	2.0*	-5.6***

B

**Accession**	**GMD-2020**	**GMD-2021**
QP-002	-29.5*	17.8*
QP-003	-21.4***	15.6
QP-041	-33.0***	4.6
QP-065	-40.1***	-21.8***
QP-084	-37.4***	-24.4***
QP-089	-29.9***	-17.8***
QP-103	-29.9***	-13.6
QP-105	-25.4	-24.3***
QP-108	-26.5***	-15.3
QP-127	-49.0***	-17.0
QP-165	1.5	-12.0***
QP-220	-26.0***	11.0
QP-231	-36.5***	-23.6***
QP-346	-30.5***	-28.2***

C

**Accession**	**GMD-2020**	**GMD-2021**
QP-003	-9.2*	3.8
QP-005	-2.5***	-2.1***
QP-035	5.8***	-4.8***
QP-065	-11.2**	-5.4
QP-127	-3.5	-4.1**
QP-343	-4.5*	-2.5
QP-346	-3.1	-5.8***

D

Accession	GMD-2020	GMD-2021
QP-006	-0.5	-1.2*
QP-026	-1.2*	-1.3*
QP-030	-1.4**	-1.3*
QP-097	-0.2	-1.6***
QP-099	0.2	-1.2*
QP-105	0.7	-1.3*
QP-128	-0.4	-2.3***
QP-141	-2.4***	-0.9
QP-165	-0.8	-2.3***
QP-175	-1.9***	-2.3***
QP-176	0.2	-1.3*

E

Accession	GMD-2020	GMD-2021
QP-002	-0.1	0.2**
QP-003	0.0	0.3***
QP-004	0.1***	-0.2**
QP-006	0.2	0.1***
QP-026	0.3***	0.3***
QP-030	0.2	0.1***
QP-036	0.1**	0.0
QP-043	0.0	0.2***
QP-046	0.1	0.1***
QP-055	0.2	0.3***
QP-060	-0.2	0.3*
QP-084	0.2**	0.1***
QP-086	0.2*	0.3***
QP-096	0.2	0.2***
QP-103	0.4**	0.1***
QP-105	0.3***	0.1
QP-113	0.3*	0.2***
QP-141	0.6***	0.2
QP-169	-0.1	0.2*
QP-175	0.9***	0.5***
QP-232	0.2	0.2**
QP-233	0.3	0.2***
QP-346	0.6**	0.3***

F

Accession	GMD-2020	GMD-2021
QP-006	0.4	1.1***
QP-026	0.3	1.1***
QP-035	0.6***	-0.4
QP-036	0.5**	-0.3
QP-055	1.7***	1.1
QP-084	0.7	1.7***
QP-086	1.7***	1.9**
QP-097	0.0	0.5***
QP-105	1.1***	0.7*
QP-126	0.4	0.7***
QP-141	0.7***	0.6
QP-165	1.0*	1.9***
QP-175	1.4***	0.7
QP-176	1.1***	1.2
QP-233	0.2***	0.0

G

**Accession**	**GMD-2020**	**GMD-2021**
QP-003	3.3**	-0.2
QP-006	-0.8	2.0***
QP-026	-0.9	0.9***
QP-065	2.6***	1.4
QP-084	1.8***	2.6***
QP-086	1.9***	2.1**
QP-089	-0.6	1.1***
QP-103	2.3***	1.6
QP-105	3.4***	3.4***
QP-108	-1.5	0.7**
QP-127	1.6***	0.6
QP-175	3.3***	2.1***
QP-233	0.3	1.2*
QP-346	2.5***	2.6***

GMD: grand mean difference, *: p<0.05, **: p<0.01, *** p<0.001

Supplementary Table S3- Genotype of the accessions for SNPs associated with seed weight, plant height and flowering time. Haplotypes for the candidate genes are derived from a previous GWAS study (Patiranage, Rey et al., 2022).

**QP-code**	***CqRING***	***CqPP2C***	***CqGLX2-2***
QP-002	Hap7	Hap3	C
QP-003 [Ŧ]	Hap7	Hap3	C
QP-004	NA	Hap3	C
QP-005	Hap7	NA	C
QP-006	Hap7	Hap5	C
QP-019	Hap7	Hap3	C
QP-026	Hap7	Hap3	C
QP-030	NA	NA	NA
QP-032	Hap7	NA	C
QP-035	Hap7	Hap3	C
QP-036	NA	NA	NA
QP-041	NA	Hap3	C
QP-042	NA	Hap3	C
QP-043	Hap7	Hap3	C
QP-046	Hap7	Hap3	C
QP-055	Hap7	NA	C
QP-060	Hap4	Hap5	C
QP-061	Hap4	Hap5	C
QP-065 [Ŧ]	Hap7	Hap3	C
QP-084 [Ŧ]	Hap4	Hap5	G
QP-086 [Ŧ]	Hap4	Hap5	C
QP-089	Hap7	NA	C
QP-096	Hap7	Hap3	C
QP-097	Hap7	NA	G
QP-099	NA	NA	NA
QP-103 [Ŧ]	Hap7	NA	C

QP-105 †	NA	NA	NA
QP-107	Hap7	Hap3	C
QP-108	NA	NA	NA
QP-113	Hap7	Hap3	C
QP-126	NA	Hap3	C
QP-127 †	NA	NA	C
QP-128	Hap7	Hap5	G
QP-139	NA	Hap3	C
QP-141	NA	NA	NA
QP-165	NA	NA	C
QP-169	Hap7	Hap3	C
QP-172	Hap4	NA	C
QP-175 †	NA	Hap3	C
QP-176	Hap4	NA	C
QP-181	Hap4	Hap5	C
QP-220	Hap7	Hap3	C
QP-225	NA	NA	NA
QP-231	Hap2	Hap1	G
QP-232	Hap7	Hap3	C
QP-233 †	Hap6	NA	G
QP-343	NA	NA	NA
QP-346 †	Hap7	Hap3	C

†: selected accessions in the current study; HAP: haplotype; NA: sequence data not available

Supplementary Table S4- Best linear unbiased estimates (BLUEs) for total protein and amino acid content of quinoa accessions investigated in this study based on Craine et al [1].

Accessions	QP code	CF (%)	CP (%)	TAA (mg/g protein)	His (mg/g protein)	Ile (mg/g protein)	Leu (mg/g protein)	Lys (mg/g protein)	SAA (mg/g protein)	AAA (mg/g protein)	Thr (mg/g protein)	Trp (mg/g protein)	Val (mg/g protein)
Moroccan Yellow	QP-002	2.31	11.62	842.31	26.93	39.71	63.36	58.85	38.14	65.31	33.57	10.47	47.52
Bouchane-3	QP-003	3.15	12.08	842.13	27.20	39.75	60.95	58.31	38.15	65.20	33.03	10.50	46.17
PI-614889	QP-004	2.77	12.08	827.02	26.67	38.49	61.83	59.73	36.70	63.16	33.98	10.29	46.38
ICBA-Q5	QP-005	2.93	12.66	817.62	26.49	38.04	60.93	57.72	36.19	62.01	33.27	9.92	45.72
PI-614927	QP-006	3.55	12.46	829.39	26.88	38.99	58.60	59.03	36.46	62.60	32.64	10.10	44.02
E-DK-4	QP-019	3.33	11.60	847.54	27.08	38.76	59.08	59.24	36.89	62.06	33.72	10.21	44.07
Indian Quinoa	QP-026	3.35	11.70	840.37	26.50	39.29	60.73	58.05	39.18	64.41	33.14	10.62	45.97
PUC-mix-red	QP-030	3.91	14.32	824.18	27.18	39.48	59.83	55.91	37.00	63.81	31.88	9.59	44.95
Brightest-Brilliant-Rainbow	QP-032	2.85	12.14	827.06	26.32	37.15	59.19	60.06	37.53	61.06	33.53	10.43	44.92
RU-5	QP-035	3.54	12.21	853.63	27.57	38.75	59.06	59.37	36.94	63.21	33.38	10.52	44.35
Regalona	QP-036	3.76	13.31	831.87	26.74	38.36	58.34	55.52	35.99	61.99	32.26	9.71	43.55
Ames-13721	QP-041	3.70	13.21	831.20	27.02	38.44	56.94	58.74	37.60	63.32	32.14	10.63	42.91
Ames-13745	QP-042	2.96	11.92	834.81	26.66	37.92	59.51	61.50	37.68	62.02	33.13	10.79	44.48
Oro-de-Valle	QP-043	2.62	12.54	826.41	26.61	38.36	60.64	59.16	38.25	62.30	33.04	10.21	45.73
Ames-13744	QP-046	3.29	12.56	830.49	26.62	37.90	58.71	58.59	37.37	62.17	32.69	10.47	43.95
PUC-mix-green	QP-055	3.89	12.73	826.72	26.45	38.49	58.11	57.29	36.10	62.14	32.40	9.54	43.53
Ames-13743	QP-060	3.12	12.69	832.85	26.99	38.47	60.31	58.43	37.30	63.43	33.05	10.31	45.50
BO-58	QP-061	3.56	12.57	835.92	26.95	39.16	60.47	57.27	37.06	63.76	33.03	10.13	45.67
Vikinga	QP-065	4.02	13.26	841.46	27.36	38.79	61.49	52.35	37.34	64.20	32.84	9.93	46.21
EMBRAPA-Brazil	QP-084	3.97	13.79	830.25	27.07	39.96	60.56	53.98	37.92	64.77	31.81	9.71	45.08
Nde-09	QP-086	3.05	11.56	841.20	26.60	38.62	60.67	59.57	38.08	63.00	33.77	10.64	45.81
RU-2	QP-089	3.59	12.57	855.62	27.73	39.78	61.47	57.39	38.44	65.16	33.37	10.43	46.42
PI-634923	QP-096	3.76	12.74	815.90	26.56	38.81	60.18	56.81	37.24	63.32	32.47	9.99	45.20
NSL-86649	QP-097	4.39	15.18	834.27	28.20	40.13	60.14	52.09	34.62	63.88	31.84	9.07	44.91
BO-29	QP-099	4.07	13.33	843.80	27.62	39.74	59.54	55.76	37.02	64.79	32.83	9.77	44.90

BO-03	QP-103	3.34	12.16	834.96	26.68	38.94	61.33	57.08	37.30	63.05	33.70	9.85	45.59
NL-6	QP-105	4.15	13.74	830.37	27.21	38.99	59.21	54.88	36.76	63.50	32.11	9.62	44.09
BO-32	QP-107	4.05	14.74	834.46	27.99	38.95	59.19	55.21	35.85	63.00	32.00	9.88	44.66
BO-31	QP-108	3.61	13.68	840.52	27.61	39.81	60.16	55.26	37.41	65.17	32.31	10.10	45.53
Redhead	QP-113	3.13	11.65	831.98	26.26	37.64	58.30	60.98	37.86	62.07	33.21	10.85	43.97
BO-30	QP-126	3.44	11.84	855.52	27.12	40.23	60.37	56.63	37.66	65.57	32.89	9.88	45.11
Bouchane-4	QP-127	3.71	14.38	833.48	27.28	39.60	60.35	54.00	37.86	64.25	31.94	9.85	45.38
PI-614883	QP-128	4.39	14.77	840.81	28.20	40.14	59.68	52.41	35.65	64.46	31.73	9.14	44.25
NSL-91567	QP-139	3.39	12.88	838.84	27.24	39.87	60.53	57.02	37.96	64.94	32.78	10.23	45.64
PI-634921	QP-141	3.50	12.69	828.76	26.61	38.61	59.10	57.65	37.54	62.91	32.75	9.96	44.39
BO-51	QP-165	3.88	13.69	840.03	27.96	40.83	62.52	55.26	37.42	65.96	32.85	10.16	47.13
D-11889	QP-169	3.05	12.37	822.48	26.57	38.79	61.53	57.80	37.15	62.95	33.66	9.96	46.22
PI-634919	QP-172	3.69	12.47	841.37	27.19	39.23	59.92	58.94	36.73	63.11	33.13	10.35	44.82
BO-63	QP-175	4.46	16.43	828.15	27.63	39.69	58.83	52.52	35.13	63.29	31.42	9.06	43.70
BO-42	QP-176	3.47	12.12	842.13	26.94	38.98	59.73	58.77	37.96	63.47	33.45	10.62	45.00
BO-11	QP-181	3.84	15.02	823.85	27.34	38.99	60.16	54.66	35.92	62.83	31.90	9.56	45.05
PI-634918	QP-220	3.02	12.87	842.64	27.18	38.66	60.30	57.25	36.35	62.82	32.91	10.16	45.61
Cherry-Vanilla	QP-225	3.05	11.24	842.00	26.59	38.53	60.14	60.44	39.45	64.20	33.88	10.67	45.50
Bouchane-2	QP-231	3.20	13.80	829.77	26.61	38.38	59.60	58.23	37.73	62.75	32.65	10.34	44.75
Bouchane-1	QP-232	3.05	12.98	825.96	27.16	39.05	61.18	55.74	38.54	64.64	32.00	10.25	45.71
ICBA-Q3	QP-233	3.46	13.04	844.28	27.30	39.38	59.07	58.00	37.55	63.80	32.44	10.26	43.80
PI-614886	QP-343	NA	NA	NA	NA	NA	NA	NA	NA	NA	NA	NA	NA
Titicaca	QP-346	3.54	12.83	829.69	27.02	38.38	59.29	56.22	36.92	62.92	32.32	9.76	44.59

CF: Crude fat content, CP: Crude protein content, TAA: Total amino acid content, His: Histidine content, Ile: Isoleucine content, Leu: Leucine content, Lys: Lysine content, SAA: sulfur amino acids content, AAA: aromatic amino acids content Thr: Threonine content, Trp: Tryptophan content, Val: Valine content, NA: data not available

Craine, E.B.; Davies, A.; Packer, D.; Miller, N.D.; Schmöckel, S.M.; Spalding, E.P.; Tester, M.; Murphy, K.M. A comprehensive characterization of agronomic and end-use quality phenotypes across a quinoa world core collection. *Frontiers in Plant Science* **2023**, *14*, 1101547.

Supplementary Table S5- The daily average temperate and precipitation for the experimental period in years 2020 and 2021 in Traventhal.

Date	Precipitation (mm)- 2020	Precipitation (mm)- 2021	Average Temperature (°C)- 2020	Average Temperature (°C)- 2021
04-14	0	0	5,2	3,8
04-15	0	0	9	4,7
04-16	0	0	8,8	6
04-17	0	0	7,8	7,9
04-18	0	0,1	8	9
04-19	0	0	7,2	9
04-20	0	0	8,7	9,1
04-21	0	0	10	7,6
04-22	0	0	10	7,1
04-23	0	0	12,2	7,9
04-24	0	0	11,5	6,9
04-25	0	0	10,1	5,9
04-26	0	0	6,9	4,6
04-27	0	0	11,3	5,4
04-28	4,3	0,1	10,5	8,7
04-29	2,5	9,8	8,9	7,2
04-30	12,1	0	10,7	8,2
05-01	4,3	0,8	9,5	8,2
05-02	2,3	3,6	9,2	7,4
05-03	0	2,1	9,8	7,3
05-04	0	4,8	8,8	7,9
05-05	0	16,9	8,1	6,3
05-06	0	0	8,8	6,5
05-07	0	1,7	10,5	5,3
05-08	0	4,9	11	8,7
05-09	0	0	13,9	18

05-10	1,3	12,5	14,6	18,5
05-11	0	1,4	7,4	11,8
05-12	2,8	1,4	6,6	12,4
05-13	3,5	4,9	6,1	10,5
05-14	0	2,1	7,4	8,9
05-15	2,1	6,3	7,1	9,4
05-16	0	11,7	10,4	10,7
05-17	0	5	11,8	11,1
05-18	0	0,1	13	10,7
05-19	0	2,1	13,7	9,9
05-20	0	0,1	12,6	11
05-21	0	6	14,3	11,4
05-22	7,7	2,7	15,2	10,3
05-23	1,4	0,9	13,2	10,6
05-24	0,1	1	12,4	13,9
05-25	0	4,6	12,5	10,6
05-26	0	3,8	13,1	10,4
05-27	0	2,7	13,9	11
05-28	0	0	13	11,2
05-29	0	0	13,8	11,8
05-30	0	0	14	12,5
05-31	0	0	14,3	15
06-01	0	0	15,3	15,5
06-02	0	0	17,9	15,3
06-03	1,2	2,4	17,3	18,4
06-04	5,7	0	14	19,2
06-05	5,8	0	12	19,5
06-06	0,4	1,3	11,3	17
06-07	0,1	0	12,8	18,8
06-08	0	0	13,5	18,6

06-09	0	0	13,8	18,9
06-10	1,7	0	15,6	18,9
06-11	2,4	0	15	19,2
06-12	1	0	16,2	16,4
06-13	10,6	0	15,8	15,3
06-14	0,7	0	16,6	18,7
06-15	0	0	16,9	16,7
06-16	0	0	18,5	18,3
06-17	0	0	20,7	25,6
06-18	0,3	0	18,5	25,2
06-19	0,5	2,7	17,3	24,7
06-20	0	12,7	17,4	20,6
06-21	0	10,2	18,5	14,3
06-22	0	0	18,8	14,9
06-23	0	0	17,9	16,2
06-24	0	0	19,2	16,6
06-25	0	0	21,3	16,3
06-26	0	0	22,7	18,3
06-27	3,1	0	22,6	19,4
06-28	5,7	0	18,6	21,4
06-29	0	11	17,6	19,9
06-30	3,3	47,4	15,4	17,6
07-01	1,3	2,5	16,9	18
07-02	4	0	16,7	17,9
07-03	3,2	0	16,2	16,9
07-04	7,5	0	16,2	18,9
07-05	3,9	5,4	17,4	17,4
07-06	3,6	0	13,9	20,4
07-07	5,4	0,2	13,3	19,2
07-08	0,2	15	13,3	19,1

07-09	19,8	4,2	11,1	18,1
07-10	9,4	0	12,3	17,8
07-11	0	0	13,4	19,2
07-12	0	0	13,9	21,3
07-13	0	0,1	15,1	22,3
07-14	6,4	3	15,1	22,2
07-15	0,1	0	15,8	22,3
07-16	0	0	15,4	22,5
07-17	0	0	17,4	21,2
07-18	0	0	18,7	19
07-19	0,3	0	20,6	15,9
07-20	0	0	16,6	16,9
07-21	0	0	14,6	17,5
07-22	0	0	14,6	15,9
07-23	0	0	16,5	16,7
07-24	0,6	1,3	17,2	20,3
07-25	2,5	0	17,9	21,2
07-26	4,4	4,2	18,1	20,4
07-27	0,1	0,3	17,4	19,9
07-28	2	3	17,9	19
07-29	1,7	0,7	15	17,3
07-30	0	0,3	15,6	18,3
07-31	0	24,6	18	16,3
08-01	0	6,9	21,1	15,8
08-02	4,1	0,4	18,3	15,3
08-03	0	0	15,8	15,8
08-04	0	1,5	15,7	15,6
08-05	0	0,2	20	17,9
08-06	0	10,5	22,4	18
08-07	0	4	23	16,3

08-08	0	2,1	24	16,5
08-09	0	5,5	23,6	16,7
08-10	0	8	24,2	15,4
08-11	0	0	23,7	17,1
08-12	0	0	22,9	19,9
08-13	0	0	22,4	19,8
08-14	0	1,5	21,8	16,8
08-15	0	7,4	23	17,4
08-16	0	8,8	23,4	15
08-17	0,9	3,8	22,3	14,5
08-18	0	1,2	21	15,5
08-19	0	1,3	19,5	16,6
08-20	0,1	1,7	22,2	15,5
08-21	3,4	0	21,1	15,7
08-22	1	2,9	19,1	15,5
08-23	1,6	0	17,1	16,7
08-24	8,4	0	14,7	15,3
08-25	5,2	1,9	15,2	13,8
08-26	18	0,6	16,4	14,3
08-27	0,1	5,4	15,5	15,3
08-28	0,1	5,9	15,3	14,8
08-29	0	1	15,6	15,6
08-30	0,2	0	16,3	16,3
08-31	4,2	0	15	16,5
09-01	0,1	0	13,4	17
09-02	0	0	14,9	14,3
09-03	8,6	0	13,7	15,1
09-04	2	0	16,7	14,5
09-05	4	0	14,4	14,1
09-06	3,4	0	13	14,6

09-07	0	0	14,3	15,6
09-08	0	0	16,4	16,8
09-09	4	0	16,6	18,1
09-10	0	19,4	12,8	17,3
09-11	0	3,9	13,8	18
09-12	0,4	0,1	13	17,3
09-13	0	0	15,4	14
09-14	0	0	17,7	14,4
09-15	0	11,1	19,7	15,6
09-16	0	2,8	16,5	15,7
